# The Prophylaxis of Venereal Diseases

**Published:** 1917-12-01

**Authors:** 


### THE PROPHYLAXIS OF VENEREAL DISEASES.

### An Offence Against the General Interest.

It is no longer a social offence to talk or write about venereal diseases in fashions calculated to attract the attention of the general public. From the influence of various circumstances there has been established a general conviction that the subject is too important to permit the maintenance of a polite silence. The welfare of individual sufferers, upon not a few of whom no moral slur can be cast, the full efficiency of our armies, the economic loss to the country produced by these diseases, and the future of the nation in so far as this is compromised by still-births and infant mortality arising from venereal infection, are considerations now brought to the full light of day, and not even the most fastidious will pretend that they can any longer be ignored. It is recognised both that the subject concerns the public interest and that public opinion is an essential factor in an endeavour to meet the evils which it involves. From this conviction has issued an educational campaign avowedly directed to the teaching of the nation, and one large result of this has been the disappearance of the view that to use public money for the treatment of venereal diseases is ethically wrong and equivalent to a premium upon vice. Even those who view without sympathy the sufferings and terrors of the individual victim recognise that he must be cared for if only for the protection of his fellows. Hence with general consent, and at the cost of the community, facilities for prompt and efficient treatment have been established, and our voluntary hospitals are taking their share in the enterprise.

But while the treatment of these diseases, and especially their early treatment, has made a successful appeal to the general interest, limited sections of the community have at the same time heard that there exist certain special methods by which a still greater triumph—namely, prevention—is, at least in a large measure, possible. The most radical of all preventive measures—namely, the suppression or avoidance of illicit sexual intercourse—is, of course, an obvious suggestion, but even the most hopeful moral reformer hardly regards this measure as within the scope of practical politics so far as the present or anything like an early future is concerned. Lectures and pamphlets pointing out the terrible nature and consequences of venereal disease, appeals to personal dignity and moral sanctions, and more strict measures of police will, it is hoped, persuade or help individuals to avoid the risk of infection; but, when all is said and done, the plague, it is recognised, will continue its course in spite of these efforts and without any sensible let or hindrance. Indeed, it seems to be accepted as inevitable that the return of peace and the disbandment of our armies will now, as on former occasions, mean a large increase in the number of the victims. Thus the question naturally arises, Are there no other preventive measures? If there are, why are they not put into operation?

When these questions are put—as appropriately they are put—to the medical profession there is no difficulty in supplying confident answers to them. There are without doubt other methods of prevention, and methods, too, which, if not absolutely infallible, have been proved by actual practice to carry a very large measure of success. It is known that certain simple medicinal applications applied before exposure to risk is incurred are in very large measure protective, and were this truth widely and authoritatively proclaimed no one doubts that the incidence of venereal diseases would be largely, and even very largely, reduced. If any preventible disease other than syphilis and gonorrhœa stood in this position there would be no doubt about the issue. The protective information would be widely circulated and the public would be urged to put it into practice. The medical profession, the Government and municipal bodies, and the Churches would all alike join in the crusade, for not even in the last-mentioned of these organisations does there still linger the conviction that disease is an expression of the Divine wrath which it is our duty to endure with meekness. There is, in other words, a general acceptance of the formula that if disease is preventible the effort to prevent it is a moral and ethical duty, as well as a practical and common-sense obligation. But venereal diseases admittedly are preventible, or largely preventible, by an open proclamation of the simple and inexpensive proceedings to which allusion has already been made. Why, then, is this open proclamation not converted into a practical policy? The answer is that in the view of many—doctors as well as laymen—the issue is one not of medicine merely, but also of morals, and so far this school of thought holds the field. The Royal Commission on Venereal Diseases evaded the issue, and the Council of the Society organised for combating these diseases has declined to encourage publicity. Against this decision there have been protests by individual members of the profession both in the professional and lay journals, but up to the present the voice of the majority, or at least the vocal majority, has enforced the policy of silence and reserve.

The question, let it be freely admitted, is a difficult one, and it demands serious discussion. Earnest and competent men, and women also, are to be found on each side of the debate, and the occasion is not one for passion or intolerance. Rather clear thinking and cool judgment are desirable, and so far it is pleasant to say the discussion has been marked by these qualities. If on one side some of the speakers have had the courage to risk misrepresentation and unpopularity, representatives on the other side have shown a capacity for wide charity without any weakening of their cherished convictions, and both alike are moved by worthy motives and praiseworthy ambitions.

It will be well to review briefly the position as

THE PROPHYLAXIS OF VENEREAL DISEASES—(*continued*).

it presents itself to the opposing camps. The difference between them is limited to a single point. Both allow, of course, that illicit sexual intercourse is an unhappy and an evil practice, and that attempts to diminish it are most desirable. Both agree that educational and moral influences have some saving value and that they ought to be continued and pressed. Both admit that by suitable medical applications the risk of infection can be much reduced. Both advocate the encouragement of public facilities for early treatment. So far there is common ground. But now the ranks part company. The one party urges that measures of education and police have been tried in a more or less vigorous manner for ages, and have been tried without success. There remains the hard fact that venereal diseases and the disastrous consequences which attend them continue to exist and that the customs from which they arise show no sensible abatement. There is thus a physical ill in the community, and as this physical ill can be largely suppressed by medical means the problem is one of public health or preventive medicine. Further, the problem is of extreme simplicity. Make known the protective method in the widest possible fashion, just as is proclaimed the virtue of vaccination as a protection against smallpox. Even if as a result of this knowledge some persons, who would otherwise abstain, are led into illicit intercourse, the gain in the shape of lessened disease would far outweigh such a development, and in any event the fear of consequences has not proved an effective deterrent; those who are stayed by it are few in number, nor can they be regarded as having high moral worth. It has been plainly written by one of the exponents of these views that the suppression of venereal diseases must be regarded as a medical problem, and that by the abolition of these diseases the world will be made neither more moral nor less moral, while another distinguished member of the medical profession, writing in a lay journal, has energetically argued that the medical measures now in question should be "promoted" by the Public Health Authorities and "frankly adopted" in the Services, an expenditure of public money to be sanctioned for these ends.

The case in opposition to these claims has advanced many details, but has not always succeeded in finding a secure principle of justification. From various quarters it has been urged that sexual continence is a possibility, is compatible with health and is the true aim to be encouraged; that the protective measures proposed are not absolutely reliable; that further processes of education may yet secure larger success; that a guarantee of security will increase the number of offenders against sexual purity; that the recently provided opportunities for efficient treatment have hopeful prospects, seeing that they lessen the chances of communicating the disease; and that sexual vice is condemned by the highest sanctions. These propositions are not without value in the argument, although some of them are obviously opportunist and provisional. But none of them, at least so it seems to us, meets fully the claim that the position as regards morals will not be affected by the public or personal advocacy of medical preventive measures. This claim in substance is: The evil of illicit sexual intercourse will exist whatever you do; it must be accepted as inevitable, and the only course is so far as possible to rob it of its dangers by all known methods; in so far as the question is one of morals the teaching of methods of prevention will not be affected either one way or the other.

Is there any sufficient answer to this definition? Personally we think there is. We find the answer not so much in the position of the individual who proposes to incur risk as in that of the individual who is asked to help him to incur this risk in safety. This, indeed, is the essence of the debate. The question is not whether a person knowing of protective measures shall use them, but whether someone else shall help an ignorant and intending, or possibly intending, offender to the knowledge of these measures. No one defends the loose liver. It is the accessory or accomplice whose position is in question. Now, judged by the very lowest standard, illicit sexual intercourse is a social offence—that is, it carries or risks certain anti-social consequences. In some measure it restrains or opposes marriage. It may mean the birth of an illegitimate child, and the consequences to the individual and the community which this event implies. In practice it means the degradation of a number of women and the establishment in the community of a parasite class. Even its less coarse exhibitions may invade the sanctities of families and break the happiness of peaceful households. Admittedly it leads to experiences which offend the public conscience and injure the civic value of individuals. Leave venereal diseases and their pathological and economic penalties out of the question, the counts of the indictment here stated are still standing. Established against any individual, the offender is condemned as guilty of conduct opposed to the general interest. Allow, if you will, that the offence has always existed and probably always will exist. Its quality is not changed by the admission. Whether society provides or does not provide punishment the offence is recognised, and no one dares to challenge the verdict. For the moment, however, we are only indirectly concerned with the principal agent. What we are asked to do is to admit that it is a praiseworthy, or at least a defensible, thing to facilitate his enterprise, for to assure him that he can embark on this with safety is to facilitate his enterprise. It may be said that he will take this step in any event. Perhaps so, but in the one case we are art and part with him, while in the other we have no hand in the matter. Here, it seems to us, is the safe position for those who decline to accept responsibility for an open announcement that, given the use of certain medical applications, illicit sexual intercourse can be pursued without dread of

**THE PROPHYLAXIS OF VENEREAL DISEASE**—(*continued*).

disease. Such intercourse, whether it can or cannot be suppressed, whether it is or is not deprived of pathological danger, is prejudicial to the interests of the community, and to ask anyone to minister to the convenience of an intending offender is to ask for the assumption of the position of an accessory before the act. If this is true when the request is addressed to an individual citizen it is equally true when addressed to the community as a whole. For open announcements by local authorities or by the Legislature the citizens whom these bodies represent are responsible, and they have the power to enforce their views. In our judgment, whether the individual acts in his private or in his public capacity he has a strongly entrenched position when he declines to spread information that will be of direct service to those who will employ it in an undertaking opposed without question to the general interests of the community. The argument, as we state it, rests, it will be noted, on no lofty or supernatural sanction. It takes things as they are and claims only the tests of a morality which is purely civic, conventional, and mundane. There is, of course, another basis for morality, but with this at the moment we are not concerned. As individual citizens or as members of the medical profession we are asked to circulate certain information. We decline the invitation because the acceptance of it would involve us in an offence against the general interest.

Perhaps it will be said that the adoption of the position here stated ought logically to lead to a refusal to help any person who has actually committed the offence in question. With this we do not agree. Admit, let us say, that the offence has been committed. But the community provides no penalty for it. What the community does not order for punishment no private citizen can have the right to penalise. Now, to say to an infected, or possibly infected, individual that he shall be deprived of medical treatment is to order punishment for him. If such denial is to be prescribed it must be prescribed by the Legislature. For the doctor, the offender after the event is merely a sick man, or more or less likely to become one. As such, until the law orders otherwise, he is entitled to medical succour, for it is no duty of the medical profession to judge of the moral deserts or civic values of their patients. Moreover, there are the interests of others to be considered. The infected person, or possibly infected person, is in higher or lower degree a danger to his fellows, and the treatment of the individual has thus the broadest possible defence. We wish to state this position in the strongest possible terms and at the same time to contend that it in no way conflicts with the refusal to appear charged with ready knowledge for the convenience of those who intend a practice opposed to the public interests. To help to organise in advance a social offence is one thing, but to provide succour for diseased humanity falls into quite another category. To decline the one restricts no liberty in reference to the other.

